# Mice Deficient in T-bet Form Inducible NO Synthase–Positive Granulomas That Fail to Constrain *Salmonella*

**DOI:** 10.4049/jimmunol.2000089

**Published:** 2020-07-17

**Authors:** Marisol Perez-Toledo, Nonantzin Beristain-Covarrubias, William M. Channell, Jessica R. Hitchcock, Charlotte N. Cook, Ruth E. Coughlan, Saeeda Bobat, Nicholas D. Jones, Kyoko Nakamura, Ewan A. Ross, Amanda E. Rossiter, Jessica Rooke, Alicia Garcia-Gimenez, Sian Jossi, Ruby R. Persaud, Edith Marcial-Juarez, Adriana Flores-Langarica, Ian R. Henderson, David R. Withers, Steve P. Watson, Adam F. Cunningham

**Affiliations:** * Institute of Immunology and Immunotherapy, College of Medical and Dental Sciences, University of Birmingham, Birmingham B15 2TT, United Kingdom;; † Institute of Microbiology and Infection, College of Medical and Dental Sciences, University of Birmingham, Birmingham B15 2TT, United Kingdom;; ‡ Department of Cell Biology, Center for Research and Advanced Studies, The National Polytechnic Institute, Mexico City 07360, Mexico;; § Institute for Molecular Bioscience, University of Queensland, Brisbane, Queensland 4072, Australia; and; ¶ Institute of Cardiovascular Sciences, College of Medical and Dental Sciences, University of Birmingham, Birmingham B15 2TT, United Kingdom

## Abstract

Clearance of intracellular infections caused by *Salmonella* Typhimurium (STm) requires IFN-γ and the Th1-associated transcription factor T-bet. Nevertheless, whereas IFN-γ^−/−^ mice succumb rapidly to STm infections, T-bet^−/−^ mice do not. In this study, we assess the anatomy of immune responses and the relationship with bacterial localization in the spleens and livers of STm-infected IFN-γ^−/−^ and T-bet^−/−^ mice. In IFN-γ^−/−^ mice, there is deficient granuloma formation and inducible NO synthase (iNOS) induction, increased dissemination of bacteria throughout the organs, and rapid death. The provision of a source of IFN-γ reverses this, coincident with subsequent granuloma formation and substantially extends survival when compared with mice deficient in all sources of IFN-γ. T-bet^−/−^ mice induce significant levels of IFN-γ^−^ after challenge. Moreover, T-bet^−/−^ mice have augmented IL-17 and neutrophil numbers, and neutralizing IL-17 reduces the neutrophilia but does not affect numbers of bacteria detected. Surprisingly, T-bet^−/−^ mice exhibit surprisingly wild-type–like immune cell organization postinfection, including extensive iNOS^+^ granuloma formation. In wild-type mice, most bacteria are within iNOS^+^ granulomas, but in T-bet^−/−^ mice, most bacteria are outside these sites. Therefore, Th1 cells act to restrict bacteria within IFN-γ–dependent iNOS^+^ granulomas and prevent dissemination.

## Key Points

- T-bet^−/−^ mice induce significant IFN-γ responses after *Salmonella* infection.
- Infection of T-bet^−/−^ mice enhances nonprotective Th17/neutrophil responses.
- iNOS^+^ granulomas that fail to constrain *Salmonella* are induced in T-bet^−/−^ mice.

## Introduction

Diseases caused by *Salmonella enterica* range from a mild, self-limiting gastroenteritis to severe invasive presentations, such as enteric fever and invasive nontyphoidal salmonellosis, characterized by a high risk of death (1). Postinfection, intracellular pathogens such as *S. enterica* typically enter professional phagocytes, such as macrophages, and reside intracellularly within vacuoles (2). Mouse models of invasive disease demonstrate that from these niches, bacteria can spread within the host, ultimately resulting in disease (3). Temporally, control of these infections develops in two stages. Initially, bacterial growth is limited by the innate system before clearance is mediated by adaptive immunity. Central to controlling infection and enhancing the activities of innate cells are Th1 cells and their associated cytokines such as IFN-γ (4). Such Th1 cytokines are important for controlling these infections in humans and mice (5, 6), with mice lacking IFN-γ unable to control bacterial growth and succumbing to infection within a few days of challenge (7, 8). Nevertheless, the amount of IFN-γ generated at different stages of infection is not equal. Studies in mice have shown that the level of IFN-γ produced in response to infection is significantly greater when adaptive immunity is active than during the early stages of infection when this cytokine is largely derived from innate sources (8). Therefore, IFN-γ is likely to play significant, although possibly differing, roles at early and late stages of infection.

In contrast to the rapidly fatal infections observed in IFN-γ–deficient mice, infections of mice lacking CD4^+^ T cells, with defective T cell survival, or compromised features of Th1 immunity (such as the key Th1-associated transcription factor T-bet^−/−^) reveal a more complex scenario. In such mice, IFN-γ production is reduced in CD4^+^ T cells, yet mice are able to control infection similarly to wild-type (WT) animals at the early stages of infection but ultimately lack the capacity to control it, succumbing weeks after challenge (9–12). The requirement for a functional T cell compartment is also evidenced by the increased risk of invasive nontyphoidal *Salmonella* infections in humans with HIV, with lower CD4^+^ T cell counts correlating with greater risk (13, 14). In contrast, studies have shown that CD8^+^ T cells play a modest or nonessential role in promoting bacterial clearance (15, 16). Collectively, these data suggest that to understand immune-mediated control and resolution of *S. enterica* infections, then it is essential to study the IFN-γ axis in innate and adaptive responses at different times postinfection.

A striking feature of the response to systemic *Salmonella* Typhimurium (STm) infection is that immune cells organize in tissues to form complex environments that can help to contain the spread of the bacteria (17–19). These inflammatory foci resemble granulomas and contain a range of cell types including infected macrophages, monocytic cells, neutrophils, and T cells (20). Within these foci, bacteria are typically found in association with inducible NO synthase (iNOS) cells (19). An inability to form early inflammatory foci, such as when IFN-γ is absent, is associated with an increase in bacteria numbers and dissemination (7, 20), but the contribution of immune and nonimmune sources of IFN-γ in this is unclear. Moreover, how T cells within inflammatory foci help constrain infection is not fully elucidated. For instance, although the induction of iNOS is essential for bacterial clearance and requires IFN-γ, iNOS is dispensable for the early control of infection, indicating that its activity is strongly linked to Th1 cell function (21).

Therefore, although we have a good understanding of the factors associated with successful immunity, less is known about how these factors link together longitudinally. In this study, we have used mice deficient in IFN-γ or T-bet to examine the anatomy of successful and unsuccessful immune responses in the spleen and liver by assessing where bacteria reside within infected tissues, with the immune response generated. Our results characterize how IFN-γ and T-bet interplay to fine tune the mechanisms of bacterial clearance.

## Materials and Methods

### Mice, infection protocols, and in vivo interventions

Animals were used in accordance with the Home Office guidelines at the University of Birmingham. The following mice were bred and maintained in the Biomedical Services Unit at the University of Birmingham: IFN-γ^−/−^, T-bet^−/−^, TCRβδ^−/−^, and IgHκ^−/−^ (all on C57BL/6 background and sources described in Refs. 22–25). WT controls were purchased from Charles River Laboratories. For all experiments, a mixture of male and female mice was used at 6–12 wk of age.

All infections were performed with the attenuated STm strain SL3261 as described previously (26). Mice were injected i.p. with 10^5^ CFUs per mouse. To quantify bacterial burden in tissues, portions of spleen and liver were homogenized, diluted 10-fold, and plated onto Luria–Bertani (LB) agar plates.

For IL-17 neutralization experiments, T-bet^−/−^ mice were infected as described above. Two days postinfection, mice were injected i.p. with 100 μg of anti–IL-17–neutralizing mAb (Clone 17F3; BioXCell) or rat IgG as isotype control (Sigma-Aldrich) every other day until day 14 postinfection.

### Generation of bone marrow chimeras

To generate IFN-γ bone marrow chimeras, recipient mice were treated with Baytril for 1 wk prior to irradiation. Both WT and IFN-γ^−/−^ mice were irradiated with two doses of 450 rad given 4 h apart. One hour after the final dose of radiation, each mouse was reconstituted i.v. with 10^7^ bone marrow cells from WT or IFN-γ^−/−^ mice and left to reconstitute for 10 wk before infection.

To generate chimeras that lacked T-bet specifically in T cells or B cells, recipient mice (either TCRβδ^−/−^ or IgHκ^−/−^) were put on Baytril for 2 wk and then irradiated and reconstituted as described above. Recipient mice were reconstituted i.v. with 10^7^ bone marrow cells in an 80:20 ratio of TCRβδ^−/−^ or IgHκ^−/−^ in combination with T-bet^−/−^ or WT cells. Therefore, in the generated mice, T cells (TCRβδ^−/−^ recipients) or B cells (IgHκ^−/−^ recipients) lacked T-bet whereas the remainder of cells were T-bet sufficient. For those mice that received mixed WT bone marrow cells, all cells in the recipient mice were T-bet sufficient. Mice were left to reconstitute for 10–12 wk before infection.

### Flow cytometry

Single-cell suspensions from spleens were prepared, and RBCs were lysed with ACK Lysis buffer (Life Technologies). Liver single-cell suspensions were prepared as described previously (20). Cells were stained for viability using Zombie Dyes (BioLegend) prior to incubation with primary Abs for 30 min at 4°C. For intracellular cytokine detection, 3–5 × 10^6^ cells were incubated overnight in anti-CD3–coated wells (clone 145-2C11, 10 μg/ml; eBioscience) in the presence of anti-CD28 (final concentration 1 μg/ml; clone 37.51; eBioscience) and Golgi Stop (BD Biosciences). Intracellular staining was carried out using the Foxp3 Fixation/Permeabilization Kit (eBioscience) following the manufacturer’s instructions. Data acquisition was performed on a Fortessa LSR II analyzer (BD Biosciences) using the FACSDiva6.2 software (BD Biosciences), and data were analyzed with FlowJo Software v10.5 (Tree Star). A list of all Abs used can be found in Supplemental Table I.

### Histology

Acetone-fixed, 5-μm cryosections of spleens and livers were stained for immunohistochemistry as described previously (27). Briefly, sections were rehydrated in TBS (pH 7.6) and incubated with primary Abs at room temperature for 45 min. The primary Abs used their sources and concentrations can be found in Supplemental Table I. HRP-conjugated or biotin-conjugated secondary Abs and ABComplex Alkaline Phosphatase (Dako) were used to detect labeled cells. HRP activity was detected with SIGMA*FAST* 3-3′Diaminobenzidine Tablets, whereas alkaline phosphatase activity was detected using Naphtol AS-MX Phosphate and fast blue salt with levamisole (all from Sigma-Aldrich). Staining for immunofluorescence was performed as described (28). Briefly, sections were rehydrated in PBS (pH 7.4) and incubated with primary Abs in the dark at room temperature for 40 min. Secondary Abs were used in all cases and were added for 30 min at room temperature in the dark. The details of primary and secondary Abs used are in Supplemental Table I. After staining, slides were mounted in Prolong Diamond (Invitrogen).

### Cytokine quantification

Cells obtained from spleens and livers were resuspended in RPMI-1640 medium supplemented with 10% FCS (Sigma-Aldrich) and 1% penicillin/streptomycin (Life Technologies). A total of 5 × 10^5^ cells were plated in 96-well plates precoated with anti-CD3ε (clone 145-2C11; eBioscience), and anti-CD28 (clone 37.51; eBioscience) was added at a final concentration of 1 μg/ml. For cell stimulations using heat-killed STm, STm SL3261 was grown in LB broth and washed twice in PBS before heat inactivation (75°C for 1 h, with killing confirmed by plating onto LB agar plates) and addition to plates at 10^7^ killed bacteria per well. Cells with medium only were included as nonstimulated controls. Cells were incubated at 37°C with 5% CO_2_ for 48 h. Supernatants were harvested and aliquots were stored at −80°C until analysis. Cytokines were detected by ELISA following the manufacturer’s instructions (mouse IFN-γ and IL-17 Ready-Set-Go Kits; eBioscience).

### In vitro neutrophil phagocytosis assay

Neutrophils were enriched from the bone marrow of WT or T-bet^−/−^ mice by density gradient centrifugation as described in (29). The purity of the neutrophils obtained by this method was >80%. To infect neutrophils, 5 × 10^5^ enriched neutrophils were plated in a 96-well U-bottom plate, and STm that express GFP or mCherry (30, 31) added at multiplicities of infection from 0.1 to 10. Plates were incubated at 37°C with light agitation for 2 h. After this, the cells were washed and incubated for 1 h in RPMI 1640 containing 50 μg/ml of gentamicin. Cells then were washed and stained for flow cytometry analysis as described above. Data acquisition was performed on a CytoFLEX Flow Cytometer (Beckman Coulter) using the CytExpert software version 2.4 (Beckman Coulter), and analyzed with FlowJo Software v10.5 (Treestar).

### Image analysis

Images were acquired on a Zeiss Axio Scan.Z1 Microscope with a 20× lens objective and analyzed using Fiji software. The proportion of pixels in a field of a particular fluorochrome was calculated by using the Renyi’s entropy algorithm to allow autothresholding on single-channel images (32). The number of F4/80^+^ foci was calculated by applying the “Analyze particles” tool on autothresholded images. Particles between 0.005 and 1 inch^2^ were counted. At least five different fields were analyzed per tissue per mouse, and results reported as the average of these. Numbers of CD3^+^ cells per focus were quantified by point counting on images viewed on Zen software (Blue Edition). At least 100 foci were counted for each section, and the average number of CD3^+^ cells per focus is reported. Numbers of *Salmonella* per unit of section was quantified by point counting as described previously (28). Median fluorescence intensity (MFI) was obtained by selecting at least 200 foci per liver section per time-point per condition across multiple mice.

### Statistical analysis

Statistical analysis was performed using GraphPad Prism 8 (GraphPad software). Mann–Whitney *U* test, two-tailed nonparametric one-way ANOVA with Dunn post hoc test, or two-tailed nonparametric two-way ANOVA with Tukey post hoc test were applied. In all cases, statistical difference between groups was considered significant at *p* < 0.05.

## Results

### T-bet in T cells is essential to clear STm

**FIGURE 1.**
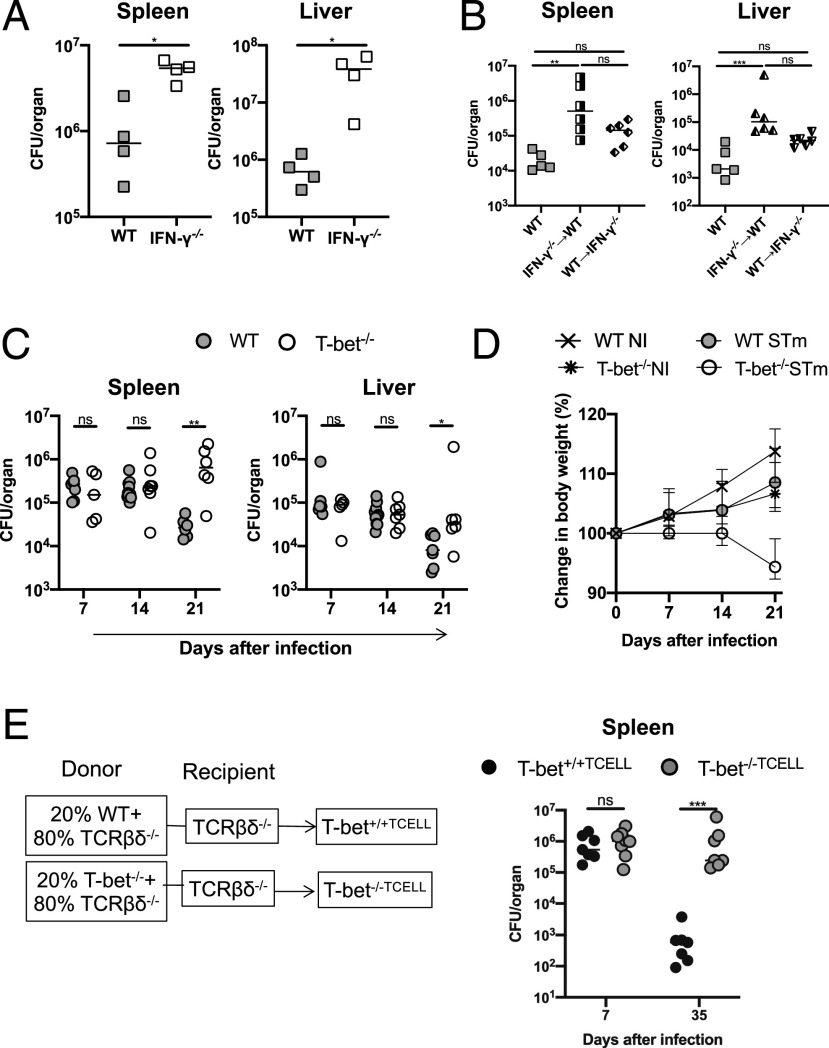
T-bet in T cells is essential to control STm infection after i.p. infection with 10^5^ STm SL3261 cells. (**A**) Bacterial burdens in the spleen and liver of WT and IFN-γ^−/−^ 7 d postinfection. (**B**) Bacterial burdens in the spleens and livers of IFN-γ chimera mice 21 d postinfection. The *x*-axis shows the donor into recipient. (**C**) Bacterial burdens in spleens and livers of WT and T-bet^−/−^ mice at days 7, 14, and 21 postinfection. (**D**) Changes in body weight in the mice from (C). (**E**) Bacterial burdens in the spleens of mice with T-bet–sufficient T cells (T-bet^+/+TCELL^) or T cells lacking T-bet (T-bet^−/−TCELL^) on days 7 and 35 postinfection. Each point represents a value for the tissue of a single mouse, and bars show the median value, except for (D) in which the median weights and SEM of mice are shown. Mann–Whitney *U* test was applied in (A), (C), and (E); nonparametric one-way ANOVA with Dunn post hoc test was applied in (B). *p < 0.05, **p < 0.01, ***p < 0.001. ns, nonsignificant.

The role of IFN-γ in protection against STm infection is well established (7). An i.p. infection of WT mice with 10^5^ attenuated strain STm SL3261 was used in all the studies described below and results in a systemic but nonlethal infection that starts resolving by day 21 postinfection. However, infection in IFN-γ^−/−^ mice results in a poorly controlled infection, with bacterial numbers 10-fold greater or more in the spleen and liver by 7 d postinfection (Fig. 1A), and for welfare reasons the experiment needs to be stopped at or shortly after this time, otherwise the mice will die. To examine whether any source of IFN-γ can contribute to survival, we generated IFN-γ chimeras by irradiating WT mice or IFN-γ^−/−^ mice and reconstituting with bone marrow cells from IFN-γ^−/−^ or WT mice respectively, and infecting for 21 d. In addition, we reconstituted IFN-γ^−/−^ mice with IFN-γ^−/−^ bone marrow cells and infected, but these experiments were stopped at 7 d postinfection, for welfare reasons (Supplemental Fig. 1A). Although there were some differences in bacterial numbers between the groups in different organs, the key finding was that both sets of chimeric mice were able to survive until at least day 21 postinfection, a time-point that is not possible in this model using IFN-γ^−/−^ mice. Thus, the provision of a source of IFN-γ was enough to confer some control of the infection.

Previous work examining the role of T-bet in STm infection has shown that splenic CD4^+^ T cells from T-bet^−/−^ mice exhibit defective production of IFN-γ postinfection (9). To examine this in more detail, WT and T-bet^−/−^ mice were infected with STm for up to 21 d. On days 7 and 14, both WT and T-bet^−/−^ had similar bacterial burdens in the spleen and liver (Fig. 1C). However, on day 21, T-bet^−/−^ mice showed significantly higher bacterial burdens in both organs compared with WT mice (Fig. 1C), with a greater fold difference observed in the spleen than in the liver. Accordingly, T-bet^−/−^ mice lost more weight, reflective of a worsening condition (Fig. 1D). Using combinations of WT, T-bet^−/−^, TCRβδ^−/−^, and IgH^−/−^ mice, we made chimeras in which T-bet was lacking in T cells or B cells. These mice were then infected for up to 35 d. These experiments showed that all of the mice controlled the infection similarly at day 7, but that T-bet was essential only in T cells for clearance at later time points (Fig. 1E). In contrast, chimeras that lacked T-bet in the B cell compartment only controlled the infection equally well as mice with T-bet–sufficient B cells (Supplemental Fig. 1B). These results indicate that T-bet is required to control STm infection at late time points, and that T-bet in T cells is necessary for this.

### T-bet^−/−^ mice have reduced IFN-γ production in CD4^+^ T cells but not in CD8^+^ T cells

**FIGURE 2.**
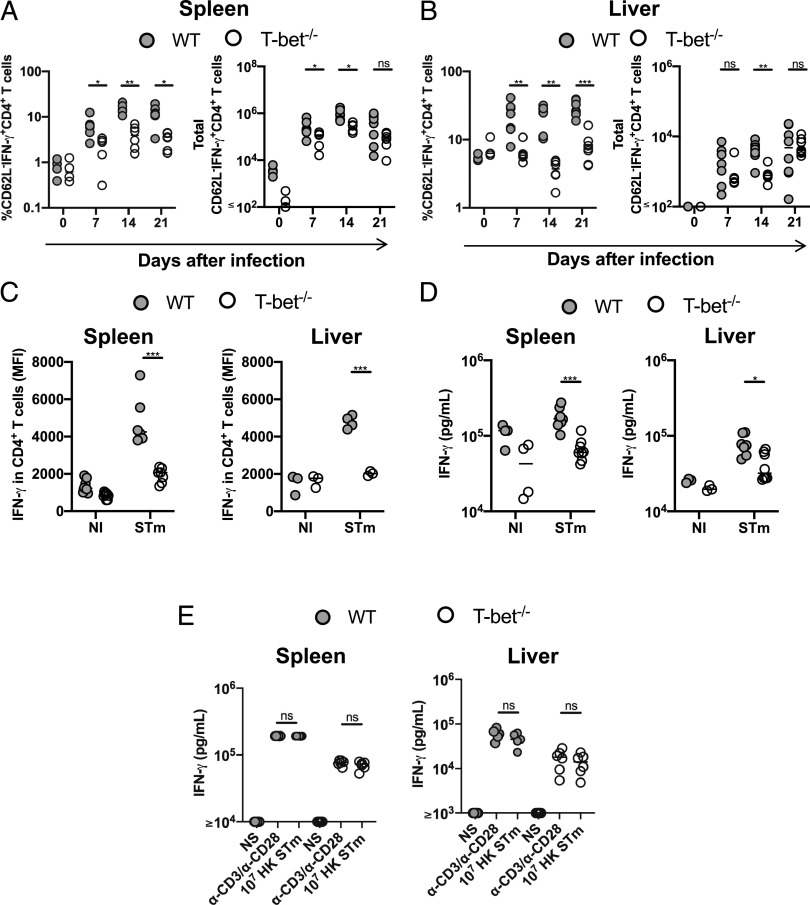
Differential dependence on T-bet for IFN-γ production in CD4 and CD8 T cells post–-STm infection. Frequency and total numbers of IFN-γ^+^CD62L^−^CD4^+^ T cells in (**A**) spleen and (**B**) liver. (**C**) MFIs of IFN-γ in CD62L^−^CD4^+^ T cells from spleen and liver on day 21 postinfection. (**D**) Quantification of IFN-γ in supernatants from cells obtained from spleens and livers on day 21 postinfection stimulated with anti-CD3 and anti-CD28. (**E**) Quantification of IFN-γ in supernatants from spleen and liver cells obtained from mice infected for 21 d: nonstimulated (NS), stimulated with anti-CD3 and anti-CD28, or stimulated with 10^7^ heat-killed STm (HK). Each point represents a value for the tissue of a single mouse, and bars show the median value. Data combined from at least two independent experiments. Mann–Whitney *U* test was applied in (A) and (B). Nonparametric two-way ANOVA with Tukey post hoc test was applied in (C) and (D). *p < 0.05, **p < 0.01, ***p < 0.001. ns, nonsignificant.

We then examined the production of IFN-γ and TNF-α in T cells, which are key Th1-associated markers. Longitudinal analysis of IFN-γ and TNF-α production in CD4^+^ T cells in the spleens and livers of infected T-bet^−/−^ mice showed that TNF-α was induced normally, whereas IFN-γ was not (Fig. 2, Supplemental Fig. 1C, representative flow cytometry shown in Supplemental Fig. 1D–G). At each time-point postinfection, lower frequencies of IFN-γ^+^ CD4^+^ T cells were found in the spleens and livers of T-bet^−/−^ mice compared with WT mice (Fig. 2A). This typically corresponded to fewer IFN-γ^+^ CD4^+^ T cells in the spleen, but this difference was less pronounced in the liver (Fig. 2B). However, the MFI for IFN-γ was significantly lower in T-bet^−/−^ CD4^+^ T cells, which indicates that those IFN-γ^+^ CD4^+^ T cells detected produced less IFN-γ (Fig. 2C). Based on these results, we anticipated that we would detect substantially reduced amounts of IFN-γ in restimulated splenocytes or purified leukocytes from the liver. Surprisingly, the amount of soluble IFN-γ was only 50% lower compared with WT controls in the spleen and liver (Fig. 2D), and this was also observed when cells were restimulated with killed bacteria (Fig. 2E). This suggested that there were other cellular sources producing IFN-γ independently of T-bet.

**FIGURE 3.**
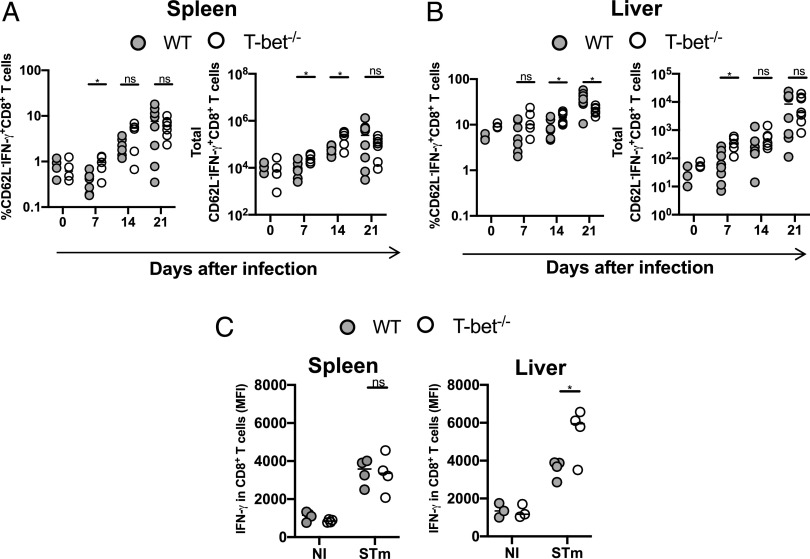
IFN-γ production is maintained in CD8 T cells in the absence of T-bet. Frequency and total numbers of IFN-γ^+^CD62L^−^CD8^+^ T cells in (**A**) spleens and (**B**) livers postinfection. (**C**) MFIs of IFN-γ in CD62L^−^CD8^+^ T cells from spleens and livers 21 d postinfection. Each point represents a value for the tissue of a single mouse, and bars show the median value. Data combined from at least two independent experiments. Mann–Whitney *U* test was applied in (A) and (B). Nonparametric two-way ANOVA with Tukey post hoc test was applied in (C). *p < 0.05. ns, nonsignificant.

Consistent with this, in the spleen, proportions and numbers of IFN-γ^+^CD8^+^ T cells were similar or slightly greater (Fig. 3A), and a similar result was observed in the liver (Fig. 3B). Furthermore, the MFI for IFN-γ in T-bet^−/−^ CD8^+^ T cells was similar or higher than WT CD8^+^ T cells (Fig. 3C). Therefore, although the absence of T-bet results in defective CD4^+^ Th1 responses, TNF-α production is still maintained in these cells and CD8^+^ T cells are still able to produce significant levels of IFN-γ after STm infection.

### Th17 responses are enhanced in T-bet^−/−^ mice after STm infection

**FIGURE 4.**
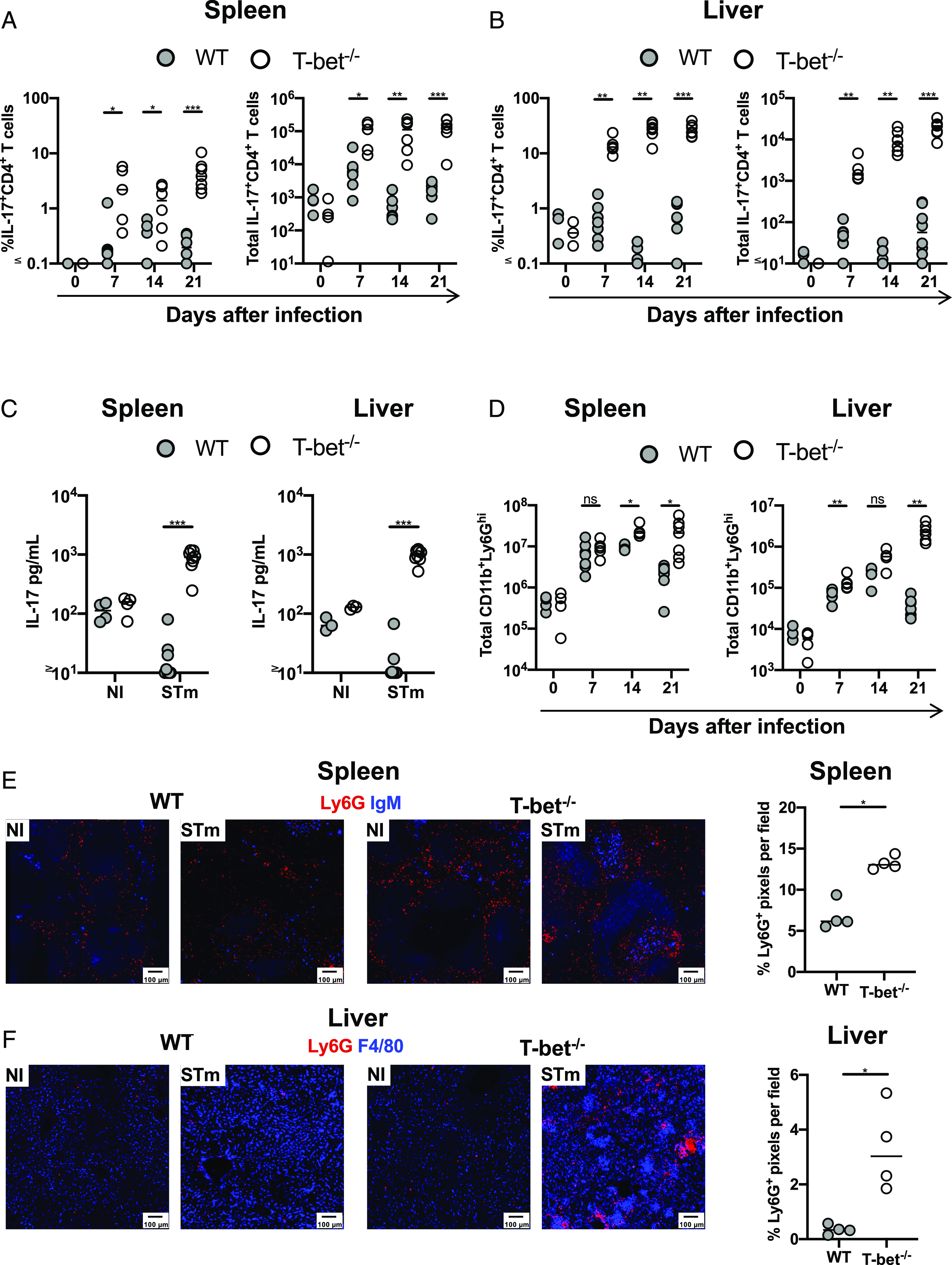
Th17 responses are enhanced in T-bet^−/−^ mice after STm infection. Frequency and total numbers of IL-17^+^CD62L^−^CD4^+^ T cells in (**A**) spleens and (**B**) livers from mice infected with STm. (**C**) Quantification of IL-17A in supernatants from cells obtained from spleens and livers 21 d postinfection. (**D**) Total numbers of CD11b^+^Ly6G^hi^ (neutrophils) in spleens and livers at different times postinfection. (**E**) Representative immunofluorescence micrographs of a spleen (E) and liver (**F**) from WT and T-bet^−/−^ mice that were noninfected or infected for 21 d. Graphs show the percentage of Ly6G pixels detected per field. Spleen sections stained with anti-Ly6G (red) and anti-IgM (blue) and livers with anti-Ly6G (red) and anti-F4/80 (blue). Each point represents a value for the tissue of a single mouse, and bars show the median value. Data combined from at least two independent experiments with three to five mice per group. Mann–Whitney *U* test was applied in (A), (B), and (D). Nonparametric two-way ANOVA with Tukey post hoc test was applied in (C). *p < 0.05, **p < 0.01, ***p < 0.001. ns, nonsignificant.

The absence of T-bet is associated with enhanced Th17 cell development (33). In WT mice, the frequency of IL-17^+^ CD4^+^ T cells in the spleen and liver before and postinfection was ≤1% (Fig. 4A, 4B). In contrast, in T-bet^−/−^ mice, there was a dramatic increase in proportions and numbers of IL-17^+^ CD4^+^ T cells, which was apparent from day 7 postinfection, such that at day 21 there were >100-fold more IL-17^+^ CD4^+^ T cells in T-bet^−/−^ mice than WT mice (Fig. 4A, 4B, representative plots in Supplemental Fig. 1F). We also found significantly higher levels of IL-17 in the supernatants of spleen and liver cells obtained from T-bet^−/−^ (Fig. 4C). Th17 responses are linked to granulocyte recruitment, and in T-bet–infected spleens and livers, there was an increase in the numbers of neutrophils postinfection (CD11b^+^Ly6G^+^ cells; Fig. 4D–F). In the spleen, the neutrophils were scattered throughout the red pulp (Fig. 4E), whereas in the liver, most were located in the sinusoids or surrounding blood vessels (Fig. 4F). These results suggest that in the absence of T-bet, Th17 responses are enhanced and are associated with a concomitant neutrophilia.

### Neutralizing IL-17 reduces neutrophilia but does influence bacterial clearance

**FIGURE 5.**
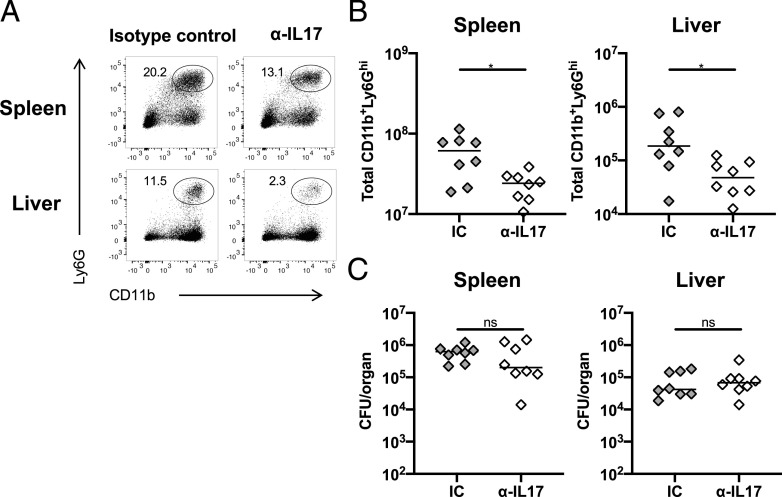
Neutralizing IL-17 reduces neutrophilia but not bacterial numbers. (**A**) Representative FACS dot plots of CD11b^+^Ly6G^hi^ (neutrophils) in spleens and livers of T-bet^−/−^ mice infected with STm for 14 d and treated with isotype control (IC) or anti–IL-17A–neutralizing Ab from the time of infection. (**B**) Total numbers of neutrophils and (**C**) bacterial burdens in spleens and livers of infected mice treated with the neutralizing Ab or the IC as described in (A). Each point represents a value for the tissue of a single mouse, and bars show the median value. Data combined from two independent experiments with four mice per group. Mann–Whitney *U* test. *p < 0.05. ns, nonsignificant.

As neutrophils can suppress effective adaptive immune responses (34), we examined whether the failure to control infection in T-bet^−/−^ mice was due to this accumulation of neutrophils. First, we tried direct depletion of neutrophils using Abs. However, use of both anti-Ly6G and anti-Gr1 Abs in infected T-bet^−/−^, but not WT mice on day 14 resulted in adverse clinical signs within an hour after administration and the experiment needed to be stopped. Therefore, an alternative approach was undertaken, whereby an anti–IL-17–neutralizing Ab was administered to T-bet^−/−^ mice throughout the infection. Neutralizing IL-17 resulted in a significant reduction in the numbers of neutrophils in the spleen and liver (Fig. 5A, 5B) but had no impact on bacterial numbers in these organs (Fig. 5C). Therefore, neither IL-17 nor increased neutrophil numbers contribute to the impaired protection against STm observed in T-bet^−/−^ mice.

### Loss of T-bet does not result in defective induction of inflammatory foci

**FIGURE 6.**
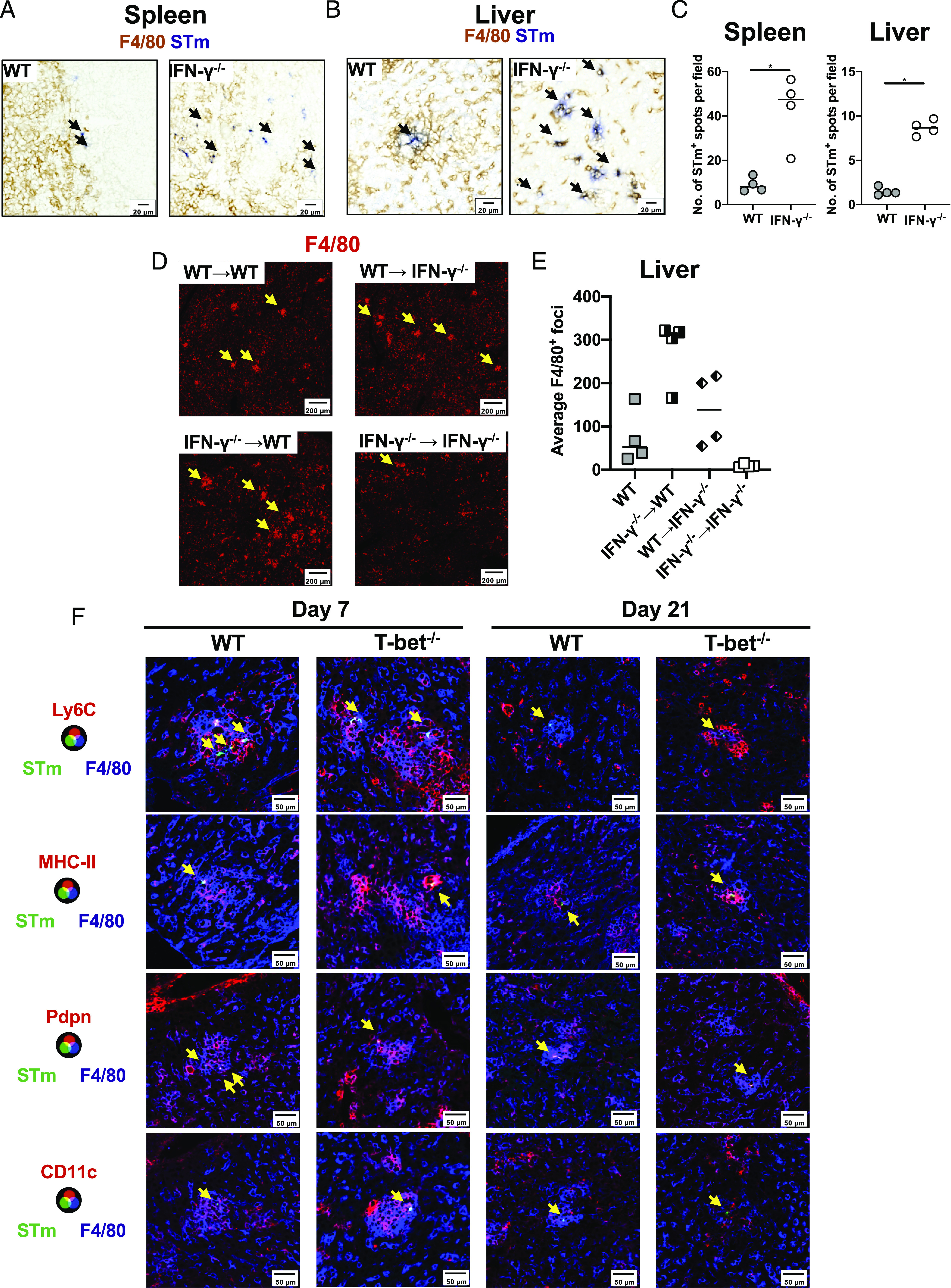
STm infection induces inflammatory foci in T-bet^−/−^ mice. Representative immunohistochemistry micrographs of (**A**) spleen and (**B**) liver cryosections from WT or IFN-γ^−/−^ infected for 7 d with STm. F4/80 is shown in brown and *Salmonella* (STm) in blue. Black arrows indicate bacteria. (**C**) Quantification of STm per field from (A) and (B). Mann-Whitney *U* test. *p < 0.05. (**D**) Representative immunofluorescence micrographs of liver cryosections stained with anti-F4/80 (red) from IFN-γ chimeras 7 d postinfection. Yellow arrows indicate inflammatory foci. (**E**) Average number of F4/80^+^ foci per field from (D). (**F**) Representative immunofluorescence micrographs of liver cryosections from WT or T-bet^−/−^ mice infected for 7 (left) or 21 d (right) stained for F4/80 (blue), STm (green) and Ly6C (first row), MHC-II (second row), Pdpn (podoplanin, third row), and CD11c (fourth row). Yellow arrows indicate the location of bacteria.

Postinfection, STm are rapidly restricted within inflammatory foci that contain multiple cell types, including F4/80^+^ monocytic cells, granulocytes, and T cells (19, 20). We therefore assessed whether such structures form and contain bacteria postinfection. Inflammatory foci were not discernible in IFN-γ^−/−^ mice and there is widespread dissemination of bacteria throughout the tissues, with bacteria typically associating with single F4/80^+^ cells, or more occasionally, very small clusters of F4/80^+^ cells (Fig. 6A–C). In the IFN-γ chimeras, there was some capacity to induce inflammatory foci in all of the chimera combinations except for chimeras that lacked all sources of IFN-γ (Fig. 6D, 6E). Because the provision of a source of IFN-γ resulted in foci formation, we hypothesized that the reason T-bet^−/−^ mice fail to control infection is not due to an inability to form such structures. Inflammatory foci formed in T-bet^−/−^ and resembled those of WT mice (Fig. 6F, Supplemental Fig. 2E). Within foci, the cellular makeup was similar in both groups of mice, with both being rich in F4/80^+^ cells and cells expressing Ly6C, MHC class II (MHC-II), CD11c, and the marker of activation podoplanin (Fig. 6F). In all the mice examined, bacteria were more commonly associated to F4/80^+^ cells and much less with neutrophils. Even in T-bet^−/−^ mice, the frequency of STm associated to Ly6G^+^ cells was around 10–15% in the spleen (Supplemental Fig. 2A), potentially explaining why decreasing the number of neutrophils did not affect bacterial numbers. This is unlikely to be because neutrophils cannot be infected, as in vitro studies showed STm-infected neutrophils from WT and T-bet^−/−^ mice to similar levels (Supplemental Fig. 2B, 2C). Therefore, although the induction of inflammatory foci is IFN-γ–dependent, this cytokine can be sourced from multiple cellular niches. Moreover, the diminished IFN-γ response observed in CD4^+^ T cells in T-bet^−/−^ mice and the consequent increased bacterial burdens are not associated with a failure to generate inflammatory foci.

### STm associates with iNOS^+^ cells at a lower frequency in T-bet^−/−^ mice

**FIGURE 7.**
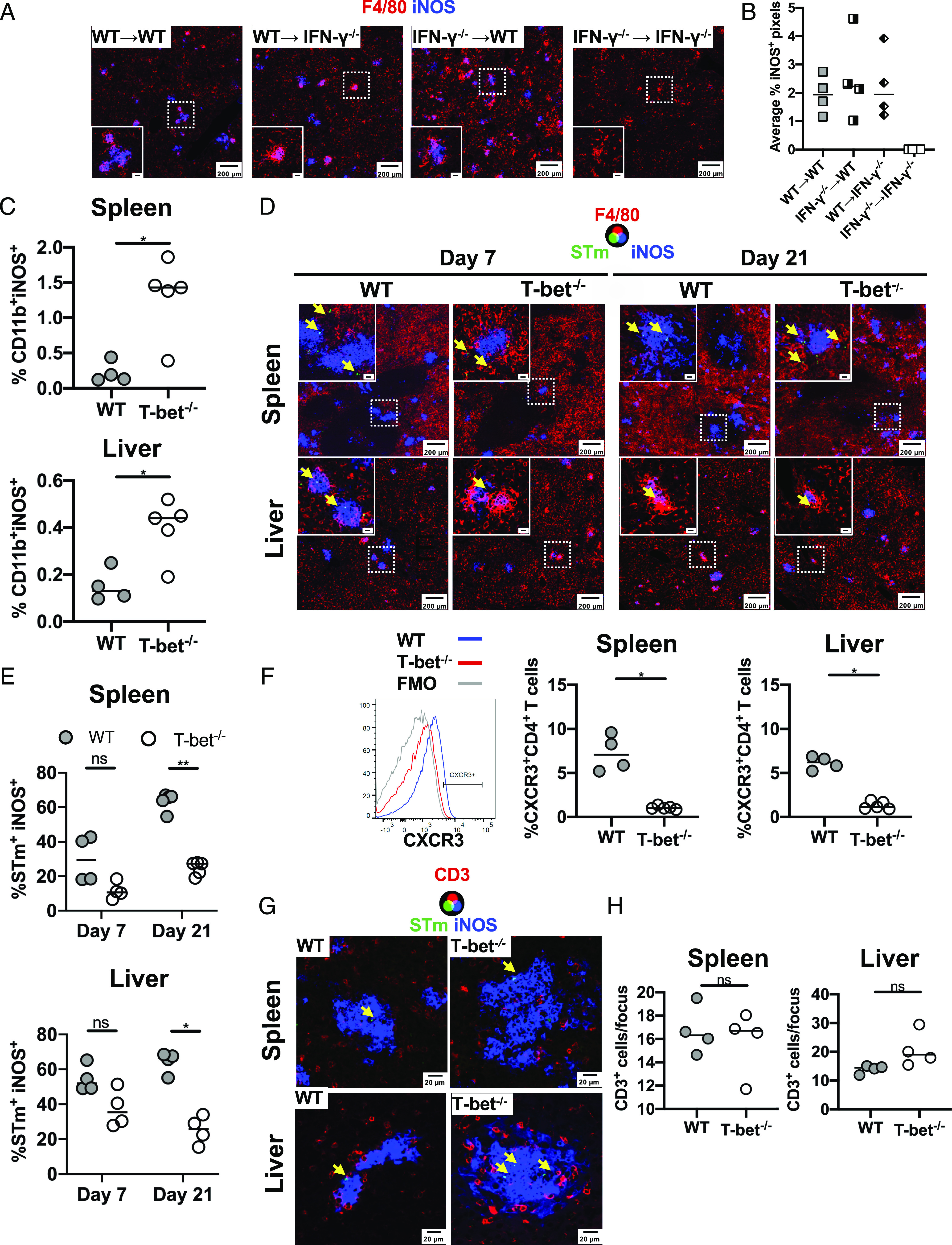
STm associates less with iNOS^+^ cells in T-bet^−/−^ mice. (**A**) Representative immunofluorescence micrographs of liver sections from IFN-γ chimera mice infected for 7 d. Sections were stained for F4/80 (red) and iNOS (blue). Scale bar in the inset is 20 μm. (**B**) Percentage of INOS^+^ pixels per field from (A). (**C**) Frequency of CD11b^+^iNOS^+^ cells determined by FACS from the spleen and liver of WT and T-bet^−/−^ mice infected for 21 d. (**D**) Representative immunofluorescence of spleens (top row) and livers (bottom row) from WT and T-bet^−/−^ mice infected for 7 (left) or 21 d (right) stained for F4/80 (red), iNOS (blue), and STm (green). Yellow arrows indicate the localization of bacteria. (**E**) Frequency of STm associated with iNOS^+^ cells per section from infected spleens and livers. (**F**) Frequency of CXCR3^+^CD4^+^ T cells in spleens and livers of WT and T-bet^−/−^ mice on day 21 postinfection. Representative histograms of CXCR3 expression on CD4^+^ T cells in the spleen of WT (blue line) or T-bet^−/−^ (red line) 21 d postinfection are shown on the left. (**G**) Representative immunofluorescence micrographs from cryosections of spleens and livers from WT and T-bet^−/−^ mice infected for 21 d. Sections are stained for CD3 (red), iNOS (blue), and STm (green). Yellow arrows indicate the localization of bacteria. (**H**) Average number of CD3^+^ cells per iNOS^+^ focus from (G). Data are expressed as medians. Mann–Whitney *U* test. *p < 0.05, **p < 0.005.

Because inflammatory foci appear similar in WT and T-bet^−/−^ mice, it suggests that defective clearance of bacteria could reside with a function of the inflammatory foci. One key molecule that is associated with clearance of infection, but not its early control, is iNOS. Therefore, we examined iNOS expression in IFN-γ^−/−^ chimeras and T-bet^−/−^ mice. This showed that in the absence of all sources of IFN-γ, iNOS is not induced in response to infection (Fig. 7A, 7B). However, the expression of iNOS was restored as long as some source of IFN-γ was available (Fig. 7A, 7B). Thus, multiple sources of IFN-γ can contribute to iNOS induction. Substantial expression of iNOS was detected in the T-bet^−/−^ mice at day 21 postinfection, with expression in the liver largely localized to inflammatory foci (Fig. 7C, 7D, representative FACS plots in Supplemental Fig. 2D). We then examined the relationship between bacterial localization and iNOS-expressing cells. At day 7 postinfection, we found that in WT mice, around 30% of bacteria in the spleen and 50% of bacteria in the liver were associated to iNOS^+^ cells (Fig. 7E). These frequencies tended to be lower in T-bet^−/−^ mice, but not significantly so. Nevertheless, at day 21, >80% of bacteria were associated with iNOS^+^ cells in WT spleens and livers, but this proportion was <25% in T-bet^−/−^ mice (Fig. 7E). Because CD4^+^ T cells surround iNOS^+^ granulomas that contain STm (19), we assessed whether the defect in bacterial containment was associated with a deficient positioning of CD4^+^ T cells. Although we found a lower frequency of CXCR3^+^ CD4^+^ T cells in both spleen and liver from T-bet^−/−^ compared with WT mice (Fig. 7F), we did not find a defect in the localization of CD3^+^ cells that surrounded iNOS^+^ granulomas that contain bacteria (Fig. 7G, 7H). Thus, in the absence of T-bet, iNOS is induced but there is a failure to contain bacteria within iNOS^+^ cells, thus leading to a protracted failure to clear infection.

## Discussion

This work examines the localization of bacteria and the immune architecture of spleens and livers from *Salmonella*-infected mice. The study focuses on a model of infection in which mice will resolve infection and two scenarios in which failed immunity will ultimately lead to host death. In the first scenario, the role of IFN-γ is examined when it is absent from all sources. In this case, the immune architecture of infected mice resembles noninfected mice despite having large bacterial burdens that are not controlled. In the second scenario, we have examined responses in T-bet^−/−^ mice which have reduced IFN-γ production from CD4^+^ T cells. In this case the appearance of the response in WT and T-bet^−/−^ mice is more similar, again, despite there being an inability to control the infection. Thus, in the first example, failure looks like the steady-state, and in the second example, immune failure resembles successful immunity. Therefore, failure at discrete checkpoints of immune control can have a distinct presentation.

A key finding from our studies and those of others is the association between successful restriction of bacterial dissemination and the formation of granuloma-like structures (18–20). In line with this, IFN-γ–deficient mice do not form foci, and in these mice, bacteria disseminate and there is rapid death. When Th1 responses are deficient, there is minimal impact in the capacity of mice to form foci, and the cells within foci express classical markers of activation. For instance, in both WT and T-bet–deficient mice, inflammatory foci contained F4/80^+^ Ly6C^+^ cells that express MHC-II, CD11c, and podoplanin. Podoplanin-expressing monocytes are more likely to express IFN-γ than their podoplanin-negative counterparts, suggesting that the innate cells present are also activated (20). As expected, bacteria associated with monocytic cells and only low numbers of bacteria could be found per cell (17, 18). Nevertheless, within an individual inflammatory focus, few bacteria were detected despite there being many monocytic cells present. Strikingly, only a small proportion of bacteria were detected in neutrophils, despite their accumulation in inflammatory foci. This was the case even in T-bet^−/−^ mice that had an enhanced neutrophilia. Indeed, it was curious that neutrophils were constituent cellular components of inflammatory foci but were rarely directly infected. The proportion of STm associated with Ly6G^+^ cells in the spleen was similar between WT and T-bet^−/−^ mice and in vitro infection assays showed a similar capacity of bacteria to infect Ly6G^+^ cells, suggesting that the neutrophils from these mice maintain at least some similar functional characteristics.

There are a number of reasons why the enhanced neutrophil numbers in the T-bet mice did not contribute to control. First, this may relate to the timing of when T-bet is important for the control of infection. WT and T-bet^−/−^ mice control infection equally well in the first week postinfection, when control is entirely independent of adaptive immunity. Therefore, because innate immunity is similar between both groups, any neutrophilia in T-bet^−/−^ mice may not augment clearance further. Second, it may be that monocytic-lineage cells are simply more efficient at capturing STm and so neutrophils, which are present in similar numbers in WT and T-bet^−/−^ mice at the time of infection, are outcompeted. Postinfection is established, how bacteria spread is unclear, but STm are typically found at low frequencies per cell (18) most frequently in macrophages (35), so de novo opportunities for neutrophils to phagocytose bacteria may be limited. Additionally, the numbers of blood-borne *Salmonella* in human typhoid and invasive nontyphoidal *Salmonella* infections (in HIV^+^ individuals) is low, typically <1 bacterium per milliliter of blood (36, 37), and even in murine models bacterial numbers in blood and lymph are modest (20, 38). Therefore, the inability of neutrophils to kill STm may reflect most bacteria being “protected” from neutrophils, despite their elevated numbers in T-bet^−/−^ mice. Third, there may be differences in the activity of different cell types at different anatomical locations. Neutrophil infiltration into the gut is a hallmark of enteritis associated with nontyphoidal *Salmonella* infections in humans. After oral infection of mice with STm there is a rapid recruitment of neutrophils in Peyer Patches and in mesenteric lymph nodes, where depletion studies (using anti–Gr-1 Abs) suggest they contribute to the early control of infection (39–42). Nevertheless, because anti–Gr-1 Abs recognize an epitope common to Ly6G and Ly6C (43), they can also deplete nonneutrophil populations too, such as monocytic cells, and this may influence the outcome of experiments (44, 45). Thus, more studies on the role of neutrophils in *Salmonella* infections are needed to evaluate the roles and activities of these cells.

In T-bet^−/−^ mice, granulomas are similar to those in WT mice, suggesting that failure is due to other downstream effector mechanisms or the ability to localize bacteria to such sites of control. Jenkins and colleagues demonstrated that STm localizes to iNOS-enriched structures, which are surrounded by CXCL9- and CXCL10-expressing cells that attract CD4^+^ T cells (19). This is a key finding because iNOS is essential for clearance at times when control is T cell dependent (21). Nevertheless, we did not find an impairment in the induction of iNOS in T-bet^−/−^ mice, meaning iNOS induction on its own cannot be enough to promote bacterial killing, indicating additional factors are required. Therefore, Th1 cells act to restrict bacteria to these sites of control and the additional levels of stimulus provided by Th1 cells promote killing downstream of iNOS expression. However, the highest proportions of STm localized to iNOS^+^ granulomas were observed in WT mice at times when control of infection is T cell dependent, indicating that localization to iNOS^+^ cells is dependent on T-bet and T cells.

The identification of a significant minority of STm not localized within iNOS^+^ sites, even in WT mice, can help explain why Th1 responses can take weeks to clear STm in this model, which has been a conundrum for the field. T cell responses develop quickly in secondary lymphoid tissue postinfection with *Salmonella* (46). In the gut, T cell activation is detected within hours postoral infection, and in other sites such as the spleen, T cell priming is also equally rapid in postsystemic challenge (47, 48). Despite this, clearance takes weeks. This is likely to reflect the capacity of the bacteria to evade sites of iNOS^+^. This could be because bacteria can occupy cellular niches resistant to the induction of iNOS. The key elements to investigate in the future are whether bacteria localize to different monocytic populations that differ in their sensitivity to T cell–promoted killing or if this is an effect of the bacteria to escape immune control. The importance of different monocytic populations has recently been examined during chronic *Salmonella* infections, with M2 macrophages correlating with a reduced capacity to control *Salmonella* infection (49).

Postinfection with *Toxoplasma gondii*, T-bet is required for optimal T cell trafficking into sites of secondary infection (50). Similar to the cited work, we also observed a reduction in the expression of CXCR3 on CD4^+^ T cells in the absence of T-bet. Although Jenkins and colleagues have proposed that the expression of CXCR3 enables CD4^+^ T cells to localize to iNOS^+^ granulomas, we found little difference in the numbers of T cells present within foci. Also, in granulomas formed in response to *Leishmania major* infection, iNOS expression does not require direct interaction between APC and CD4^+^ T cells (51), which means that IFN-γ can still induce iNOS expression even if CD4^+^ T cells are not directly interacting with the granuloma-forming cells; therefore, failure to control *Salmonella* infection in T-bet^−/−^ mice is not related to an inability of CD4^+^ T cells to migrate to infected tissues.

In infected T-bet^−/−^ mice, the induction of TNF-α was maintained in CD4^+^ T cells, whereas infection resulted in enhanced Th17 responses, which were largely undetectable in infected WT mice. The induction of TNF-α shows that T cell priming is productive in terms of enabling some features of classical Th1-associated responses other than IFN-γ. Nevertheless, the induction of TNF-α appears insufficient to promote bacterial clearance. Augmented Th17 responses in T-bet^−/−^ mice have been reported in other infectious models, with *Francisella tularensis* and influenza virus (52, 53) with variable consequences on protection in these models. Because Th17 responses are linked to a neutrophilia within tissues, and that under some circumstances these cells can mediate tissue damage (54) and impair T cells responses (34, 55, 56), we hypothesized that aberrant Th17 responses could contribute to the failure to control *Salmonella* infection. Neutralizing IL-17 reduced the neutrophilia but did not have an effect on bacterial numbers. Oral infection with *Salmonella* induces the production of IL-17, primarily from innate sources, and these play a protective role (57). However, mice deficient in IL-17 infected systemically with *Salmonella enteritidis* showed only a mild defect in bacterial control (58), suggesting that Th1 responses are sufficient to clear this infection. Complementing these findings, our results indicate that although Th17 responses are enhanced in T-bet^−/−^ mice, increased Th17 responses do not compensate for reduced Th1 activities. This indicates that there is a compartmentalization of T helper responses to *Salmonella* that provide differential protection in distinct niches, with a predominant role for Th1 responses in systemic sites and a more important role for Th17 responses at mucosal sites.

Another factor that may influence the control of *Salmonella* infection in T-bet^−/−^ mice is the effect that T-bet can have on the development of T regulatory cells, in particular a subset that expresses both Foxp3 and T-bet and that has been proposed to regulate Th1 responses (59–61). We did not examine the impact of T-bet on FoxP3^+^ T cell populations postinfection, nor its effect on any potential mediator of regulation, such as TGF-β. Recent work suggests that the depletion of T regulatory cells can alter the balance between Th1 and Th17 responses in the mucosa after oral STm infection (62). Hence, the ratio and number of FoxP3^+^ T cells within tissues may be influenced by the absence of T-bet, and this effect should be examined in depth in future studies.

The impact of STm infection in IFN-γ^−/−^ mice has been described previously and is associated with high bacterial numbers and rapid death. Although it is well described that IFN-γ stimulates iNOS expression, it is unlikely that the reason for the rapid death in these mice is because of deficient iNOS expression. For instance, the expression of NADPH oxidase and its metabolites seem to play a bigger role in controlling bacterial replication from the very first stages postinfection (63). In contrast, iNOS is only required after the first week postinfection onwards (21). Our imaging of STm-infected IFN-γ^−/−^ mice showed that there was a much wider distribution of infected cells compared with WT tissues, and that infected cells did not cluster with other monocytic cells. Thus, in the context of IFN-γ^−/−^ mice, death is more likely associated with disseminated infection and the classical endotoxemia as has been reported previously (64). In contrast, in the IFN-γ chimera mice, as long as there was a source of IFN-γ available, the mice regained the capacity to form inflammatory foci. When this happened, then bacterial dissemination was less prominent and survival was prolonged, a phenotype similar to the one observed with T-bet^−/−^. This suggests that IFN-γ can contribute to immunity in multiple ways at different times postinfection, with early IFN-γ production in the liver contributing to the rapid accumulation of immune cells to help limit bacterial spread. Future studies need to address the potential sources of the IFN-γ that help achieve this prolonged survival, as this will help discern whether different IFN-γ sources contribute to the response in different ways, such as promoting cellular recruitment or immune cell aggregation.

In conclusion, Th1 responses work at late stages of *Salmonella* infection to maintain the containment initiated by IFN-γ at the early stages, and thus result in effective bacterial clearance.

## Supplementary Material

Data Supplement
