# Supplementary material for: Mice Deficient in T-bet Form Inducible NO Synthase–Positive Granulomas That Fail to Constrain Salmonella
Source: J Immunol. 2020 Jul 17;205(3):708–19. doi: 10.4049/jimmunol.2000089 (PMC7372318; doi:10.4049/jimmunol.2000089)
Supplement: Data Supplement [file JI_2000089.zip › JI_2000089_Supplemental_Material_1.pdf]

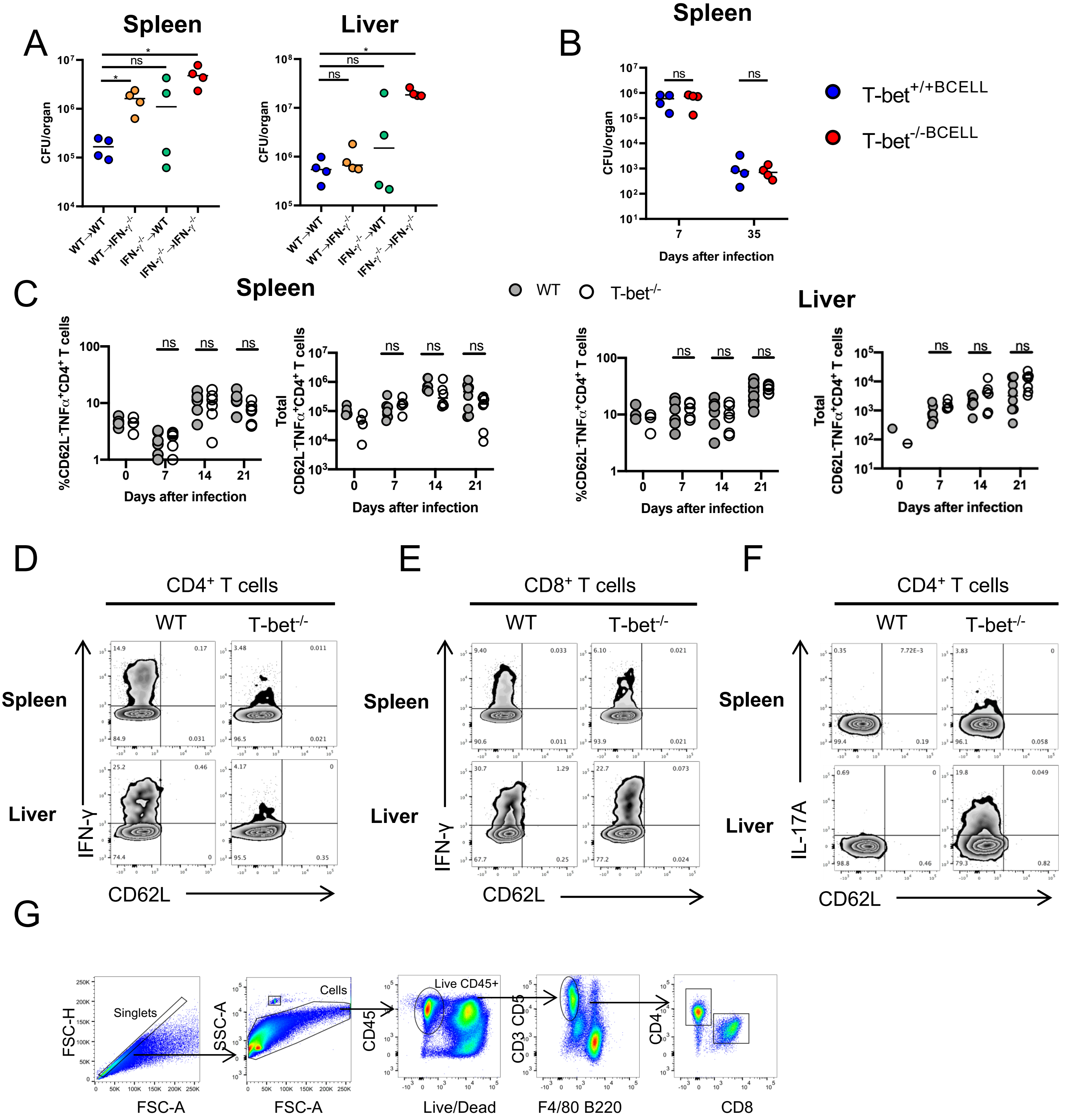

Supplemental figure 1. (A) Bacterial burdens in the spleen and liver in IFN-γ chimeras 7 days post-infection. IFN-γ chimera mice were generated as described in the methods section, and infected i.p. with 10^5 CFU for 7 days. n= 4 mice per group. Each point represents a value for the tissue of a single mouse, and bars show the median value. Mann-Whitney U test was applied. *P<0.05, ns=non-significant. (B) Bacterial burdens in the spleens of mice with T-bet-sufficient B cells (T-bet$^{+/+BCELL}$) or mice lacking T-bet in B cells (T-bet$^{-/-BCELL}$) on days 7 and 35 post-infection. Each point represents a value for the tissue of a single mouse, and bars show the median value. (C) Frequencies and total numbers of CD62L$^-$CD4$^+$TNFα$^+$ cells in spleens and livers of WT and T-bet$^{-/-}$ mice infected for 7, 14 or 21 days. Data combined from two independent experiments. Mann-Whitney U test was applied. ns=non-significant. (D-E) Representative gating to detect intracellular IFN-γ production in T cells from the spleen and liver of infected mice in CD4$^+$ T cells and (D) CD8$^+$ T cells (E). (F) Representative dot plots of intracellular IL-17A detection in CD4$^+$ T cells. (G) Gating strategies used to examine responses in CD4 and CD8 T cells.

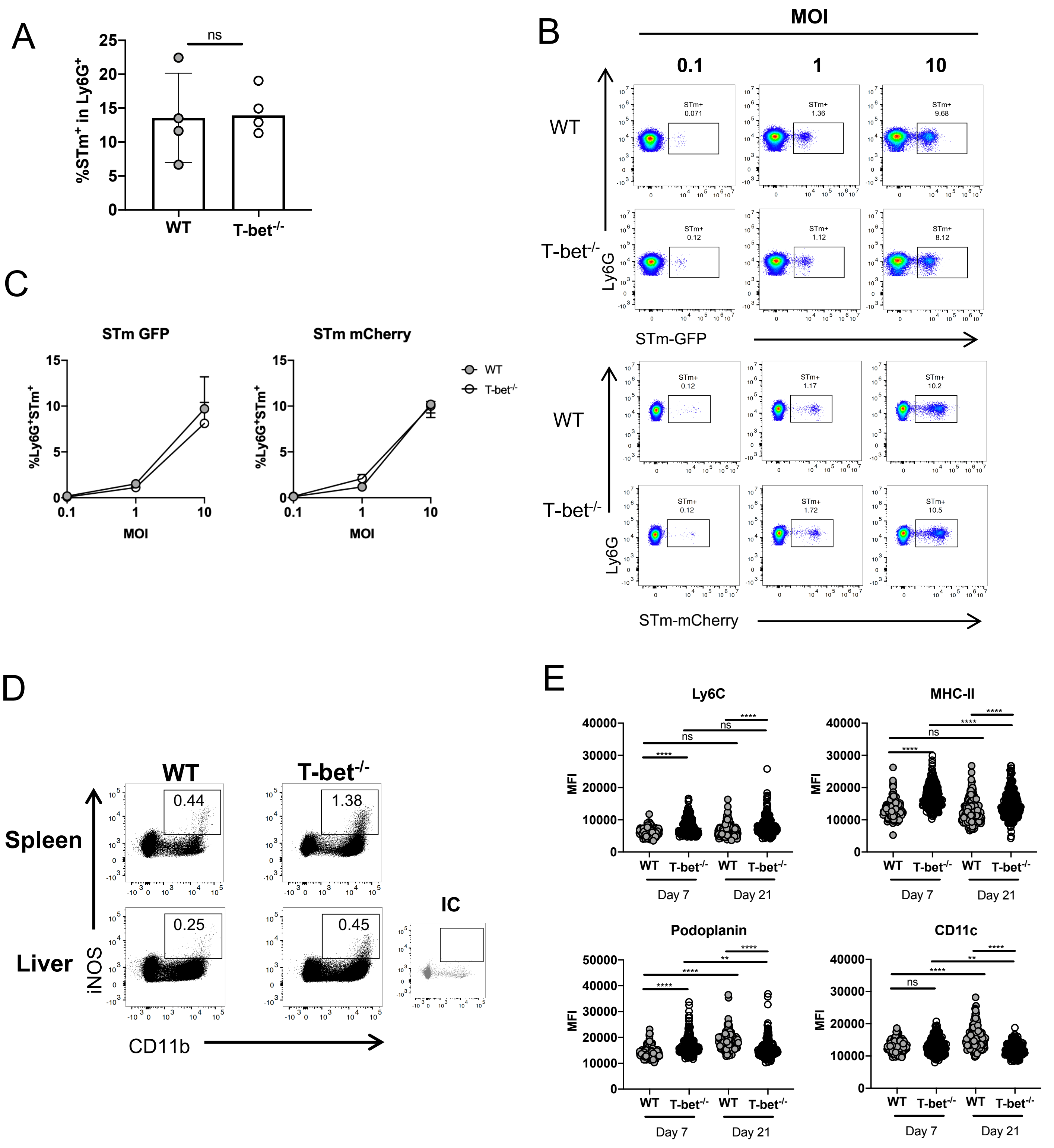

Supplemental figure 2. (A) Frequency of STm associated to neutrophils (Ly6G^{+}) cells in the spleens of WT and T-bet$^{-/-}$ mice 21 days post-infection. Cryosections from T-bet$^{-/-}$ spleens were stained for Ly6G and STm, and the frequency of STm associated to neutrophils (Ly6G^{+}) was counted. (B) Representative dot plots of *in vitro* uptake of live GFP (upper panel) or mCherry (bottom panel) STm by isolated neutrophils from WT (top row) or T-bet$^{-/-}$ (bottom row) mice. MOI: multiplicity of infection. (C) Frequencies of Ly6G^{+}STm^{+} from (B). Each point represents the median ± error of three replicates (D) Representative dot plots of intracellular iNOS staining in CD11b^{+} cells in the spleen and liver of WT and T-bet$^{-/-}$ mice infected for 21 days. (E) Liver cryosections from WT or T-bet$^{-/-}$ mice infected for 7 or 21 days stained for F4/80, STm and Ly6C, MHC-II, podoplanin and CD11c. Quantification of the median fluorescence intensity (MFI) of the given cellular marker. Representative micrographs are shown in Fig. 6F. Each point represents an individual focus. Mann-Whitney U test was applied. ****P<0.0001, ns=non-significant.

Supplemental table I. List of antibodies used in this study

**Antibodies used for Flow Cytometry**				
Reactivity	Host, isotype	Clone	Conjugate	Manufacturer
CD45	Rat IgG2b, κ	30-F11	APC-eFluor780	eBioscience
CD3	Ham Arm IgG1, k	145-2C11	PerCP-Cy5.5	BD Pharmingen
CD3	Ham Arm IgG1, k	145-2C11	PE-Cy5	eBioscience
CD3	Ham Arm IgG1, k	145-2C11	Purified	eBioscience
CD5	Rat IgG2a, κ	53-7.3	PerCP-Cy5.5	eBioscience
CD5	Rat IgG2a, κ	53-7.3	PE-Cy5	eBioscience
F4/80	Rat IgG2a, κ	BM8	Brilliant Violet 510	Biolegend
B220	Rat IgG2a, κ	RA3-6B2	Brilliant Violet 510	Biolegend
CD4	Rat IgG2a, k	RM4-5	APC	eBioscience
CD4	Rat IgG2a, k	RM4-5	Alexa Fluor 700	eBioscience
CD8	Rat IgG2a, k	53-6.7	Brilliant Violet 785	Biolegend
CD62L	Rat IgG2a, k	MEL-14	Brilliant Violet 605	Biolegend
CXCR3	Arm Hams IgG	CXCR3-173	Brilliant Violet 650	Biolegend
T-bet	Mouse IgG1, k	eBio4B10	PE-Cy7	eBioscience
Rorg	Rat IgG2a	AFKJS-9	PE	eBioscience
IFNg	Rat IgG1, k	XMG1.2	FITC	eBioscience
IL-17	Rat IgG1, k	TC11-18H10.1	Brillian Violet 711	Biolegend
CD11b	Rat IgG2b, k	M1/70	Brillian Violet 711	Biolegend
CD11b	Rat IgG2b, k	M1/70	Alexa Fluor 700	BD Pharmingen
Ly6G	Rat IgG2a, κ	1A8	PE-CF594	BD Horizon
**Antibodies used for Immunohistochemistry (IHC)**				
Reactivity	Host, isotype	Clone	Conjugate	Manufacturer
F4/80	Rat IgG2b, κ	Cl:A3-1	Purified	Bio-rad
Salmonella	Rabbit	Polyclonal	Purified	Abcam
Rabbit	Swine	Polyclonal	Biotin	Dako
Rat IgG (H+L)	Rabbit	Polyclonal	HRP	SouthernBiotech
Avidin-Biotin complex			Alkaline phosphatase	Vector Laboratories
**Antibodies used for Immunofluorescence (IF)**				
Reactivity	Host, isotype	Clone	Conjugate	Manufacturer
Ly6G	Rat IgG2a, κ	1A8	Biotin	Biolegend
IgM	Goat, IgG	Polyclonal	AMCA	Jackson Immunoresearch
F4/80	Rat IgG2a, κ	BM8	Biotin	eBioscience
Salmonella	Rabbit	Polyclonal	Purified	Abcam
iNOS	Rat IgG2a, κ	CXNFT	APC	eBioscience
MHC-II (I-A/I-E)	Rat IgG2b, κ	M5/114.15.2	Purified	BD Pharmingen
Ly6C	Rat IgM	AL-21	Biotin	BD Pharmingen
Podoplanin	Ham Syr, IgG	eBio8.1.1	Purified	eBioscience
CD3	Ham Arm, IgG	145-2C11	Purified	eBioscience
CD11c	Ham Arm, IgG	N418	Purified	eBioscience
Rabbit	Donkey, IgG	Polyclonal	Alexa Fluor 488	Jackson Immunoresearch
Rat	Donkey, Fab	Polyclonal	Alexa Fluor 647	Jackson Immunoresearch
Armenian hamster	Goat, IgG	Polyclonal	Cy3	Jackson Immunoresearch
Streptavidin			Alexa Fluor 555	Invitrogen
